# A Goal-directed Quality Improvement Initiative to Reduce Opioid Prescriptions After Orthopaedic Procedures

**DOI:** 10.5435/JAAOSGlobal-D-19-00109

**Published:** 2019-09-17

**Authors:** Kevin J. Choo, Trevor R. Grace, Krishn Khanna, Jeffrey Barry, Erik N. Hansen

**Affiliations:** From the Orthopaedic and Neurosurgery Specialists, Greenwich Hospital-Yale New Haven Health, Greenwich, CT (Dr. Choo) and the Department of Orthopaedic Surgery, University of California San Francisco, San Francisco, CA (Dr. Grace, Dr. Khanna, Dr. Barry, and Dr. Hansen).

## Abstract

Orthopaedic surgeons are increasingly aware of deleterious effects of the opioid epidemic and the association between overprescription and diversion toward nonmedical opioid use or substance abuse. Opiate prescriptions at the time of hospital discharge have been identified as target for intervention. This study describes the successful outcome of a goal-directed intervention aimed at decreasing opioid overprescription by providing routine feedback to providers regarding their prescribing patterns.

**Methods:**

The amount of opioid medications, quantified as oral morphine equivalents (OMEs), provided to opioid-naive adult patients on discharge after orthopaedic surgery was prospectively collected. As part of an institutional quality improvement initiative, medical providers received reports every 2 months detailing median discharge OMEs prescribed, trended over time. After 6 months, a retrospective comparison was done between preintervention and intervention patient cohorts.

**Results:**

There were 401 patients in the preintervention cohort and 429 patients in the intervention cohort. Both groups were similar in regard to age, sex, rates of depression, surgical time, length of stay, orthopaedic subspecialty, and inpatient opioid requirement before discharge. Patients in the intervention cohort were prescribed markedly fewer opioid medications by 25%, equivalent to 20 tablets of 5-mg oxycodone IR (450 versus 600 OMEs, *P* < 0.001). Despite these opioid medications, opioid refill rates during the first 90 days after discharge did not markedly change between groups.

**Discussion:**

It is critical to judiciously treat postoperative pain while avoiding opioid overprescription. This study demonstrated the outcome of a goal-directed initiative to decrease overprescription of opioid medications. The initiative reduced discharge opioid prescriptions yet did not increase the risk of requiring a prescription refill in the postoperative period. This indicates that such an approach can result in opioid reduction, while still providing appropriate care and pain control for patients.

Multiple studies have demonstrated that surgeons tend to overprescribe opioid medications when treating postoperative pain.^1,2,3,4,5^ When these prescription medications remain unconsumed, they represent potential for overuse and/or diversion toward nonmedical consumption.^3,6^ Indeed, previous studies have shown that most nonmedical opioids are obtained through diversion from friends and family.^6,7,8,9^ Corresponding to the rise in prescriptions, the United States has experienced a notable increase in opioid-related complications and mortalities. Between 1999 and 2015, the number of opioid-related mortalities has tripled, with an estimated 33,000 opioid-related mortalities in 2015, according to CDC data. In addition, opioid overdose is responsible for roughly 53,000 nonfatal overdose hospitalizations a year.^10^ Clearly, the overconsumption and abuse of these medications poses a notable public health and socioeconomic burden. A recent report estimated that the economic cost of nonmedical opiate use is in excess of $50 billion annually.^11^

The rise in the prescription and consumption of opioid medications in America is multifactorial, but is likely related to a historical approach to management of pain as the “fifth vital sign,” efforts to maximize patient satisfaction, and a desire to proactively manage pain.^12,13^ However, while opioid medications can be a valuable part of a post-operative pain control regimen, there is substantial evidence that excessive provision of these medications is related to the recent rise of opioid-related complications.^10^

Orthopaedic surgeons have been identified as the third highest prescribers of opioids in the United States.^12,14^ This provides a significant opportunity to improve and thereby decrease the risk of opioid-related complications in millions of patients who undergo orthopaedic procedures annually. Several approaches have been suggested to minimize opioid overuse or abuse. First, some authors have identified preoperative risk factors for overconsumption and complications after orthopaedic surgery.^15,16,17,18,19^ Second, a focus on the utilization on non-narcotic analgesia perioperatively has been endorsed as a strategy to reduce opioid exposure, ostensibly reducing the risk of chronic opioid use.^20-22^ Finally, the overprescription of opioid medications after discharge has been identified as a target for intervention.^20,22,23^

Previous studies have demonstrated that increased magnitude of opioids provided on discharge does not influence the likelihood that a patient will require a medication refill.^24,25^ Others have found notable variation and patterns of overprescription following common surgical procedures.^2^ As a corollary, decreasing the magnitude of opioids on discharge may reduce the potential for diversion without necessarily increasing the need for medication refills. Although the following may seem counterintuitive, the finding has been observed; one recent study demonstrated successful curtailment of discharge opioid medications after incorporation of consensus guidelines after elective hand surgery, without an associated rise in refills.^26^ The following is likely related to the fact that current prescribing practices far exceed the quantities actually required for adequate pain control after common orthopaedic surgeries.

Recognizing the importance of judicious opioid prescription practices, a quality improvement (QI) initiative was conducted at our institution with the goal to globally decrease discharge opioid prescription quantities by 10%. The QI project used a report sent every two months, which showed providers the median discharge oral morphine equivalents (OMEs) per patient, over time and relative to the 10% reduction goal. In addition, additional data were presented including median discharge OMEs by subspecialty and by individual provider (deidentified). The purpose of this study was to review the efficacy of the QI project implementation, in regard to both the overall quantity of opioids prescribed (in OMEs), and also assess the effect on postdischarge refill rates. The null hypothesis was that the incorporation of the intervention would not change the quantity of opioids prescribed.

## Methods

### Quality Improvement Initiative

This study was conducted at a single tertiary-care, academic hospital. As part of an institutional QI program, we sought to decrease the postdischarge prescription of opioid medications postoperatively over a 6-month period (July 1, 2017, to December 31, 2017). Specifically, the goal was to reduce the median quantity of opioid medications by 10% compared with the 6-month period before the QI initiative (January 1, 2017, to June 30, 2017). The goal reduction of 10% was determined after review by the institution's Quality and Safety office. This magnitude of reduction was believed to represent a notable improvement in responsible prescribing, without compromising postoperative pain control regimens or undertreating postoperative pain.

### Discharge Prescription Reporting

**Figure 1:**
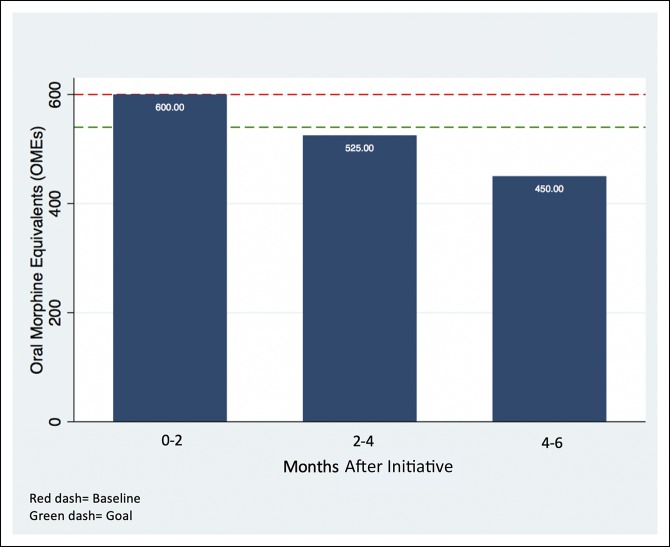
Example prescription quantity summary. Median discharge prescription (by OMEs) over time. OME = oral morphine equivalent

After obtaining Quality and Safety as well as institutional review board approval, the amount, type, and regimen of opioid medications prescribed at discharge were obtained prospectively for all opioid-naive patients undergoing orthopaedic surgery between July 1, 2017, and December 31, 2017. Pediatric patients (younger than 18 years) and patients discharged in under 24 hours were excluded. All opioid regimens were converted into OMEs. If patients were prescribed multiple opioids at discharge, each prescription was converted separately and then combined to determine the total amount of OMEs prescribed. Every 2 months, prescribers within the department (physician assistants, residents, and attending physicians) received an e-mail notification quantifying the overall median discharge opioid prescriptions per patient and summarizing the department's progress toward reaching the established goal (Figure 1). Multimodal pain control strategies, such as icing and use of acetaminophen or nonsteroidal anti-inflammatory medications, were also advocated in each e-mail to provide reinforcement regarding continued appropriate pain control management. Furthermore, additional information including median discharge OMEs per subspecialty and by individual provider (deidentified) was graphically displayed during mandatory staff meetings and grand rounds.

### Opioid Refills

Opioid prescription refills were measured at the following time points: 0 to 30 days, 31 to 60 days, and 61 to 90 days after discharge. An opioid refill event was counted during these periods if a new opioid prescription was recorded in our institution's electronic medical record (EMR)*.* The 90-day mark was determined as an appropriate end point for refills in this study because our institution's prescribing policy dictates that orthopaedic surgeons are to provide pain medications only up to the 3-month postoperative date. After that point, patients are directed to their primary care physicians for any long-term opioid medication requirements.

### Group Comparison

After the 6-month study period (July 1, 2017, to December 31, 2017), a retrospective comparison was done between patients treated during the initiative (“intervention cohort”) and patients treated the 6 months before the initiative (“preintervention cohort”). Demographic and inpatient data as well as predischarge opioid requirements, discharge prescription quantities, and postdischarge refill rates were then compared between these cohorts to characterize the effect of this opioid-reduction intervention.

### Statistical Analysis

Continuous variables (age, surgical time, length of stay, opioids consumed the 24 hours before discharge, and total opioids prescribed) were compared with the Mann-Whitney *U* test, and categorical variables (sex, history of depression, operating orthopaedic subspecialty, and opioid refills) were compared with the chi-square test. All statistical analyses were done on STATA software (version 15.0; StataCorp), with significance set to *P* < 0.05.

## Results

### Patient Cohort

A total of 1,917 opioid-naive patients required admission after an orthopaedic procedure at our single tertiary-care institution between January 1, 2017, and December 31, 2017. Pediatric patients (N = 130) and patients taking opioids before surgery (N = 957) were excluded. Thus, 830 opioid-naive adults were considered in this study: 401 patients who underwent surgery between January 1, 2017, and June 30, 2017 (“preintervention cohort”) and 429 patients who underwent surgery between July 1, 2017, and December 31, 2017 (“intervention cohort”).

### Demographic and Surgical Comparison

The preintervention and intervention cohorts were comparable in regard to age (63 years versus 63 years, *P* = 0.74), sex (51.6% male versus 52.5% male, *P* = 0.81), rates of depression (5.2% versus 4.7%, *P* = 0.70), surgical time (128 minutes versus 122 minutes, *P* = 0.77), length of stay (2.3 days versus 2.3 days, *P* = 0.95), and operating orthopaedic subspecialty (*P* = 0.91) (Table 1).

**Table 1:**
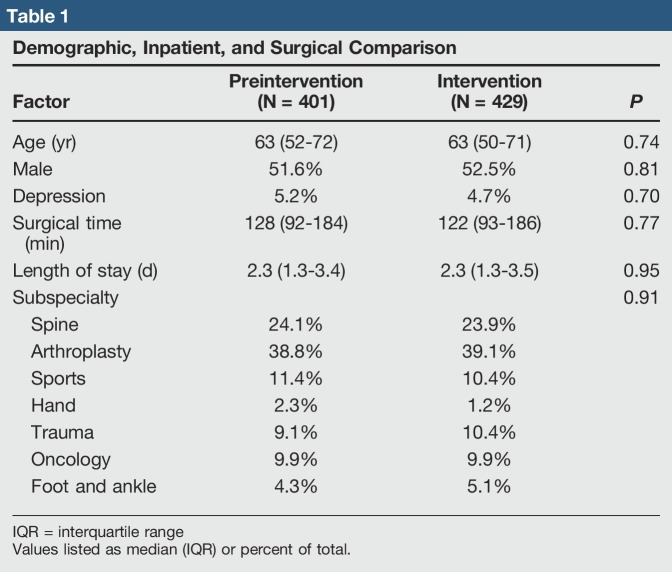
Demographic, Inpatient, and Surgical Comparison

### Prescription Comparison

**Figure 2:**
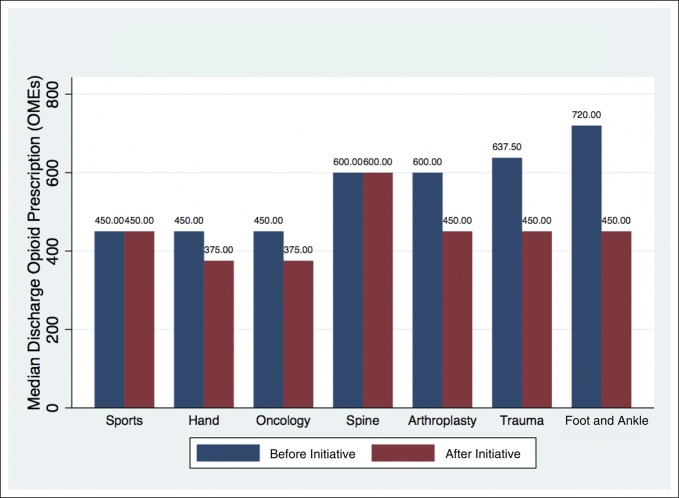
Discharge opioid prescriptions before and after initiative. OME = oral morphine equivalent

The opioid quantities required during the 24 hours before discharge were comparable between both groups (37.5 OMEs versus 40 OMEs, *P* = 0.48) (Table 2). However, the preintervention cohort was prescribed markedly more opioids at discharge than the intervention cohort (600 OMEs versus 450 OMEs, *P* < 0.001) (Table 2). The median discharge prescription quantities decreased for all orthopaedic subspecialties except Sports Medicine and Spine (Figure 2).

**Table 2:**
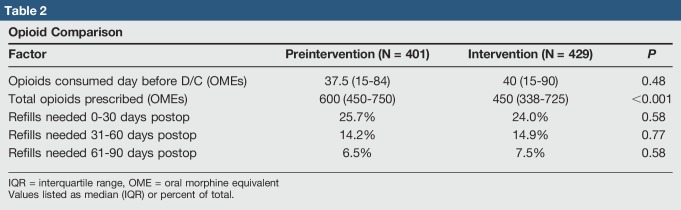
Opioid Comparison

### Refill Comparison

**Figure 3:**
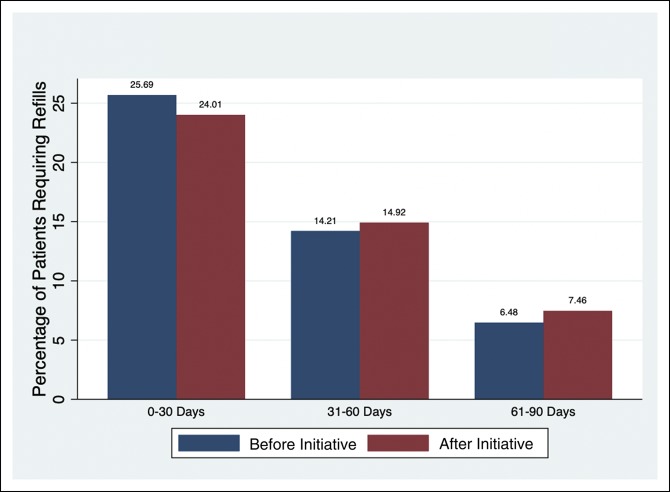
Opioid refill rates before and after initiative.

Opioid refill rates, identified from the EMR, were found to be unchanged between preintervention and intervention groups. This remained true across the three time ranges following discharge: 0 to 30 days (25.7% versus 24.0%, *P* = 0.58), 31 to 60 days (14.2% versus 14.9%, *P* = 0.77), and 61 to 90 days (6.5% versus 7.5%, *P* = 0.58) (Table 2, Figure 3).

## Discussion

The rise in opioid-related complications and mortalities has drawn interest from the highest levels of executive government and legislative bodies.^27,28^ However, despite increased social and political awareness, practitioners remain ultimately responsible for the provision of opioid medications to patients. Overprescription leading to unconsumed medications can lead to diversion, nonmedical use, and abuse. The origin of the “opioid epidemic” and its socioeconomic effect have been comprehensively chronicled.^20,28,29^

Our study demonstrated the efficacy of an institutional QI initiative to decrease the OMEs of opioid prescriptions on hospital discharge. We found that a proactive approach toward curtailing overprescription was effective in reducing the quantity of opioid medications, when comparing the intervention group with the preintervention group. Furthermore, the magnitude of reduction was substantial (450 OMEs versus 600 OMEs, *P* < 0.001). For reference, a difference of 150 OMEs translates to 20 pills of 5-mg oxycodone IR per patient. Despite a successful reduction in total OMEs prescribed on discharge, we did not observe a notable increase in the need for refills. These trends suggest that such a strategy may be effective in reducing the potential for diversion without undertreating postoperative pain. Previous studies have shown that physicians may commonly prescribe more than double the amount consumed on average after common orthopaedic procedures.^15,30,31^ The following suggests that additional reduction is achievable and appropriate without compromising postoperative care.

One facet of the intervention within the QI initiative was the use of routine updates sent through e-mail to providers every 2 months. In the update, each individual was provided information regarding recent prescribing patterns as an aggregate over time (Figure 1). This update allowed individuals to evaluate their prescribing habits and avoid outlier behavior. This observation is consistent with the principle of normative social influence, which is a type of social influence that leads to conformity. Normative influence has been recognized as a catalyst for behavior change and notably used in the energy industry as a way to reduce individual energy consumption.^32^ Here, the intervention itself simply aimed to inform providers of where they stood compared with their peers in terms of their prescribing practices. This intervention indicates that simple interventions which provide normative influence may lead to reductions in opiate prescriptions. When combined with a very clear reduction target (10%), the initiative demonstrated its efficacy in achieving its goal.

Our findings should be interpreted in the context of several important limitations. The study was done at a single academic teaching hospital in a large metropolitan area. As a result, the findings may not be generalizable to all patient cohorts or hospitals/physicians. Second, the intervention group was compared with a historical cohort (patients who underwent surgery in the 6 months before implementation of the QI initiative), and so, historical bias may be present. However, we found that there were no notable demographic differences between patient groups, and to the best of our knowledge, there were no other protocol changes to analgesia/perioperative pain management during this period which may have markedly affected findings. Generally, a multimodal approach to pain management had been protocolized across most services for years before the intervention. However, this multimodal approach has not been standardized between services. This lack of standardization may have contributed to the finding that Spine and Sports Medicine divisions did not demonstrate notable opioid reduction during the study period.

In addition, although we were able to record the provision of opioid medications on discharge and refills provided in clinic, through the use of a unified EMR, we were not able to account for opioid medications provided by an outside physician group, urgent care, or emergency department. Although we found that there were no notable differences in refill rates, we were not able to report on patient pain scores, satisfaction, or patient perceptions of their pain relief. These outcomes will be important in future studies, to ensure that decreases in pain medication prescription do not come at the cost of sensible pain management. In this study, refill rates were used as a proxy for pain control after discharge, although refill rates may be confounded by several variables such as limited patient access, hesitation to provide refill prescriptions, and opioid acquisition through diversion from friends or family.^33^

Several studies have demonstrated that current opioid prescription practices are excessive. Rodgers et al^30^, in a study of outpatient upper extremity surgery, found that their prescribing patterns were far in excess of patient consumption. In their study of 250 patients (most received 30 opioid pills on discharge), an average of 19 pills was left unconsumed, representing 4,639 leftover opioid analgesic pills available for misuse or diversion. Oxycodone, hydrocodone, and propoxyphene accounted for more than 95% of the discharge medications. Kumar et al^31^, in a study of outpatient shoulder surgeries, also found that patients had notable leftover medication. In their study, patients 90-days post-operative after shoulder stabilization/Bankart repairs had a median of 37 unused opioid pills, compared to a median of 60 prescribed at discharge. Oxycodone, hydrocodone, and codeine were the mostly commonly prescribed medications. This finding was corroborated in a study examining opioid utilization after outpatient knee arthroscopy.^15^ Ruder et al^34^ done a retrospective study of opioid-prescribing practices in a trauma cohort. They found that large quantities of opioids (calculated as OMEs) were provided to patients after discharge, including to patients believed to be at high risk of misuse and polysubstance abuse. Notably, in their study cohort, nearly one-fifth of patients were prescribed extended-release oxycodone on discharge.

In the absence of widely accepted guidelines, variability in prescribing practices will persist. Previous studies have shown that there is substantial variation in the provision of opioid medications following common surgical procedures.^35-37^ Therefore, some level of standardization may be effective, and recently, the use of prescription “maximums” has been endorsed as a possible strategy to reduce outliers in overprescription.^38^ The efficacy of standardization was demonstrated in recent study, in which the authors found that acceptance of a postoperative prescribing guideline was successful in reducing the quantity of OMEs after outpatient hand surgery.^26^ Importantly, the authors of that study found that the implementation group was not at higher risk of requiring secondary opioid medication refills. Our study extends on this finding, demonstrating that for a large number of patients across orthopaedic subspecialties, implementation of opioid reduction goals was effective in reducing opioid prescription without influencing refill rates.

Future study should focus on the development of procedure- and patient-specific guidelines to optimize prescription practices. The optimal strategy would be to provide the lowest possible dose while minimizing the need for secondary opioid refill prescriptions. Such a strategy would provide adequate analgesia for postoperative patients while decreasing the risks associated with excessive opioid use and diversion. A large study examining the “optimal” OMEs to prescribe for commonly done orthopaedic procedures would be very helpful to provide guidance to physicians managing postoperative pain. Any guideline, ideally, would also incorporate patient risk factors for higher opioid requirement/use/abuse, including previous exposure to opioids, use of long-acting narcotic medications, and/or other pertinent medical or psychosocial risk factors. In addition, the inclusion of patient pain scores, satisfaction, and perception of pain will be helpful to ensure that efforts to curb opioid overprescription do not come at the expense of adequate postoperative analgesia. These results of this study, which show a successful reduction in opioid prescriptions through the use of a QI initiative, are encouraging that such efforts can be successful without compromising care.
